# Effect of Associative Learning on Memory Spine Formation in Mouse Barrel Cortex

**DOI:** 10.1155/2016/9828517

**Published:** 2015-12-24

**Authors:** Malgorzata Jasinska, Ewa Siucinska, Ewa Jasek, Jan A. Litwin, Elzbieta Pyza, Malgorzata Kossut

**Affiliations:** ^1^Department of Histology, Jagiellonian University Medical College, 7 Kopernika Street, 31-034 Krakow, Poland; ^2^Department of Molecular and Cellular Neurobiology, Nencki Institute of Experimental Biology, 3 Pasteur Street, 02-093 Warsaw, Poland; ^3^Department of Cell Biology and Imaging, Institute of Zoology, Jagiellonian University, 9 Gronostajowa Street, 30-387 Krakow, Poland; ^4^University of Social Sciences and Humanities, 19/31 Chodakowska Street, 03-815 Warsaw, Poland

## Abstract

Associative fear learning, in which stimulation of whiskers is paired with mild electric shock to the tail, modifies the barrel cortex, the functional representation of sensory receptors involved in the conditioning, by inducing formation of new inhibitory synapses on single-synapse spines of the cognate barrel hollows and thus producing double-synapse spines. In the barrel cortex of conditioned, pseudoconditioned, and untreated mice, we analyzed the number and morphological features of dendritic spines at various maturation and stability levels: sER-free spines, spines containing smooth endoplasmic reticulum (sER), and spines containing spine apparatus. Using stereological analysis of serial sections examined by transmission electron microscopy, we found that the density of double-synapse spines containing spine apparatus was significantly increased in the conditioned mice. Learning also induced enhancement of the postsynaptic density area of inhibitory synapses as well as increase in the number of polyribosomes in such spines. In single-synapse spines, the effects of conditioning were less pronounced and included increase in the number of polyribosomes in sER-free spines. The results suggest that fear learning differentially affects single- and double-synapse spines in the barrel cortex: it promotes maturation and stabilization of double-synapse spines, which might possibly contribute to permanent memory formation, and upregulates protein synthesis in single-synapse spines.

## 1. Introduction

It is now widely accepted that behavioral experience altering the neuronal activity induces changes in the density of synapses and dendritic spines [1–3]. Synaptic plasticity has also been shown to alter synaptic efficiency by remodeling of the existing synapses [4–7].

The barrel cortex of rodents as sensory representation of whiskers as well as its afferent pathway is a useful model for studying associative learning-dependent neuronal plasticity. Classical conditioning, in which stimulation of a row of whiskers (conditioned stimulus) is paired with mild electric shock to the tail (unconditioned stimulus), changes the motor behavior of the animals and modifies the cortical representation of sensory receptors involved in the conditioning [8].

Mapping of brain activation pattern with [14C]2-deoxyglucose autoradiography showed learning-dependent expansion of functional cortical representation of the whisker row stimulated during conditioning [8]. This plasticity is associated with changes in both excitatory and inhibitory neurotransmission.

The plasticity of excitatory circuits was manifested by an increase in expression of NR2A (subunit of NMDA receptor specific for excitatory synapses) mRNA and protein [9]. In spite of that, the density of excitatory synapses or single-synapse spines did not change after conditioning [3]. However, we observed an upregulation of the number of polyribosomes associated with both excitatory and inhibitory synapses accompanied by an increase in postsynaptic density (PSD) area that suggested synaptic potentiation [7].

Conditioning also affected inhibitory transmission, inducing upregulation of GAD 67 mRNA and protein, a marker of inhibitory synapses, within the affected barrels [10], accompanied by an increase in the density of GABAergic neurons [11]. Our previous electron microscopic studies demonstrated that conditioning caused the formation of new inhibitory synapses, producing double-synapse spines in the cognate barrel hollows [3], and remodeled the morphology of double-synapse spines towards mushroom-shaped spines with shorter but thicker necks [12].

The stepwise morphological transformation of dendritic spines during their plastic remodeling that leads to formation of stable spines includes shape and size change [13, 14], acquisition of smooth ER (sER) to the spine, and formation of spine apparatus (SA) [15]. The spines containing SA are the largest [16] and it has been established that the largest spines have the longest half-life* in vivo* [17–19]. SA is a smooth ER-related membrane structure [16, 20] containing synaptopodin, a SA-specific actin-binding protein [21]. It is believed that SA is associated with the regulation of calcium storage and release [22–24] and that together with polyribosomes it can participate in the local protein synthesis [25–27]. SA is also postulated to play a role in the potentiation of synapses located on dendritic spines and in the formation of stable spines involved in memory storage and therefore called “memory spines” [23, 27, 28].

Although their function is still unknown, it seems probable that spines containing SA are involved in the synaptic plasticity [4, 29]. Inactivation of synaptopodin gene leading to the total absence of SA limited induction of long-term potentiation (LTP) and caused deficits in spatial learning [27, 30]. It was observed that fear conditioning increased the number of such spines and the number of SA-associated polyribosomes in the lateral amygdala [4].

Since the data concerning involvement of dendritic spines and spine apparatus in conditioning-induced plasticity of the somatosensory cortex are scarce, the aim of this study was to investigate the effect of short-lasting fear learning on the number and morphological features of dendritic spines in the barrel cortex by using the whisker-to-barrel pathway model and serial section transmission electron microscopy-based stereology. The barrel cortex contains two types of spines: single-synapse spines with single excitatory synapses which account for about 90% of all spines in this region and double-synapse spines with two different synapses: one excitatory and one inhibitory [12]. In each type, we separately analyzed three categories of spines, presumably representing successive levels of spine maturity: sER-free, containing sER, and containing SA.

## 2. Materials and Methods

### 2.1. Animals

The experiments were performed on Swiss Webster female mice aged 6-7 weeks, kept in standard conditions. All experiments were compliant with the Council Directive 2010/63EU of the European Parliament and the Council of 22 September 2010 on the protection of animals used for scientific purposes and approved by the Animal Care and Use Committees of the Polish Academy of Sciences and the Jagiellonian University.

### 2.2. Behavior Training

The mice (*n* = 15) were divided into conditioned group (*n* = 5), pseudoconditioned group (*n* = 5), and control, untreated group (*n* = 5). Before the onset of the conditioning procedure, all animals were habituated in a homemade restrainer which holds the mouse neck stationary, leaving the rest of the body, including the head, free. During the habituation period, mice spent 10 min per day for 3 weeks in the restrainer.

After habituation, mice were conditioned using a classical conditioning paradigm. Manual stimulation of the selected whiskers (B row; conditioned stimulus, CS) on the left side of the snout was paired with a mild electric shock to the tail (unconditioned stimulus, UCS) [8]. The pairing procedure included three sweeps back and forth along the entire whisker row with a small paintbrush lasting 3 s each, repeated at a frequency of four times per minute for 10 min, applied for 3 consecutive days. The UCS was a weak, 0.5 mA electric current applied to the tail for 0.5 s at the end of the last sweep in the series. In pseudoconditioned animals (random pairing of CS and UCS), the number and frequency of stimuli applied were the same.

### 2.3. Transmission Electron Microscopy

Twenty-four hours after completion of the conditioning, the mice were anesthetized with Vetbutal (100 mg/kg body weight; Biowet, Puławy) and perfused through the heart with 20 mL of rinse buffer (0.2% glutaraldehyde and 2% paraformaldehyde in 0.1 M phosphate buffer, pH 7.4) followed by 100–150 mL of fixative (2.5% glutaraldehyde and 2% paraformaldehyde in 0.1 M phosphate buffer, pH 7.4). The brains were removed immediately after perfusion and left in the same fixative for 24 h at 4°C.

The next day, after washing in 0.1 M phosphate buffer (pH 7.4), 60 *μ*m tangential vibratome sections were cut from the right barrel cortex. Sections were examined under a stereomicroscope (Nikon Optiphot, Japan) and those containing the barrel field cortex were collected for further processing. The sections were washed in 0.1 M cacodylate buffer (pH 7.4), postfixed twice with 1% osmium tetroxide in 0.1 M cacodylate buffer (the first time with 1.5% potassium ferrocyanide), washed in 70% ethanol containing 1% uranyl acetate, and, after dehydration in graded series of ethanol, embedded in Epon resin (Polysciences Inc., USA) between two silicone-coated glass slides.

The region of B2 and B3 barrels was trimmed for ultrathin sectioning. Series of 30 to 50 successive sections (65–70 nm thick) were cut from each sample. The sections were collected on formvar-coated copper-palladium slots and contrasted with 1% lead citrate. The central regions of the B2 barrel, layer 4, in which cell bodies are sparse and the vast majority of structures observed under TEM are dendrites, axons, and synapses were photographed at 7 K using JEOL 100SX transmission electron microscope (JEOL, Japan). We examined the collection of ultrathin sections used in our previous study [3].

Ten to twelve serial electron micrographs were taken from successive sections for 3D reconstruction of dendritic spines. The micrographs were initially aligned in Adobe Photoshop CS software, in which stacks of serial images were taken at the final magnification of 30 K.

### 2.4. Quantitative Analysis of Dendritic Spines

Quantitative analysis of dendritic spines was carried out using NIH Image J Cell Counter software (http://rsb.info.nih.gov/ij/) by placing a grid of two-dimensional sampling frame over the stack of serial sections. The dendritic spines were counted per volume unit (*μ*m^3^). Each spine was counted only once in the stack and only spines located fully within the frame or intersecting the left and the upper borderlines of the frame were included. Synapses and spines were defined according to Knott et al. [2]. The density of single- and double-synapse spines containing smooth endoplasmic reticulum (sER) and spine apparatus (SA) and sER-free spines was calculated according to the stereological formula *N*_*A*_ = Σ*Q* ^−^/*a*, where Σ*Q* ^−^ is the number of dendritic spines found in the volume *a* [31]. The counting was done blind: the observer did not know whether the micrographs were taken from conditioned, pseudoconditioned, or control animal.

### 2.5. Morphological Analysis of Spines

Serial images of 180 spines (90 single-synapse spines and 90 double-synapse spines) from control group, conditioned group, and pseudoconditioned group were selected. The selection criteria included (1) complete series of successive sections (micrographs) allowing 3D reconstruction of the spine, (2) well visible synapses, and (3) the content: SA, sER only, and none. Twelve dendritic spines meeting the above criteria, two in each group (sER-free, sER, and SA), in case of both single-synapse and double-synapse spines, were randomly selected from each animal to yield 10 single-synapse spines and 10 double-synapse spines of each category (sER-free, sER, and SA) per each experimental group (control, conditioned, and pseudoconditioned).

In every spine, length of the spine and diameter of the spine head and neck as well as excitatory and inhibitory (only in double-synapse spines) PSD areas were measured. PSD area was calculated according to Ostroff et al. [32]. Length of the spine was measured after 3D reconstruction. Spine head diameter was measured at the widest part of the head, parallel to the PSD [6]. Three measurements of the neck width at different levels were made and the mean value was calculated as neck diameter. Volume of SA was calculated by summing the values of area of SA multiplied by section thickness of all serial sections in which it appeared.

Three measurements of all parameters from every micrograph containing profiles of the selected spines were made using NIH Image J software. 3D reconstructions of the spines were performed using 3D Studio Max software (Discreet Logic, Montreal, Canada) and the location of spine apparatus (head, head/neck, or neck of the spine) as well as the number of polyribosomes in the dendritic spine was estimated.

The shapes of spines were defined according to Harris et al. [15]. Spines were divided into three shape categories on the basis of their length (*l*), diameter of the spine head (dh), and diameter of the neck (dn). Very long spines (*l* ≥ 3 × dn) with similar diameters of the head and neck (dh ≈ dn) were termed thin spines. Spine with large heads and narrow necks (dh ≥ 2.5 × dn) were termed mushroom spines. Very short spines with the length close to diameter of the neck (*l* ≈ dn) were termed stubby spines. Spines with more than one head were not observed.

### 2.6. Statistical Analysis

All data were analysed using GraphPad Prism 5.01 software (GraphPad Software Inc., USA). Differences in the densities of dendritic spines containing SA, containing sER, and sER-free as well as the SA volume across the experimental groups were analysed by Kolmogorov-Smirnov normality test and homogeneity Bartlett's test for equal variances, followed by one-way ANOVA test with* post hoc* Tukey's test. To compare the combined effect of training and spine content on the morphological measurements across the experimental groups, two-way ANOVA with* post hoc* Bonferroni test was used. Differences in shapes of spines and in the location of SA in dendritic spines between control, pseudoconditioned, and conditioned groups and cooccurrence of SA and polyribosomes were assessed by chi square test. In the text of results and in graphs, data are presented as means ± SEM.

## 3. Results

### 3.1. Sampling Areas

Dendritic spines were counted in the following total tissue volumes: control group, 484.51 ± 10.92 *μ*m^3^ (mean volume per animal 96.90 ± 7.13 *μ*m^3^); conditioned group, 457.92 ± 12.78 *μ*m^3^ (mean volume per animal 91.58 ± 7.26 *μ*m^3^); pseudoconditioned group, 479.93 ± 15.16 *μ*m^3^ (mean volume per animal 95.99 ± 8.94 *μ*m^3^). The sampling volumes were not significantly different across the groups (*F*_(1, 14)_ = 0.13, *P* = 0.88).

### 3.2. Density of Dendritic Spines

**Figure 1:**
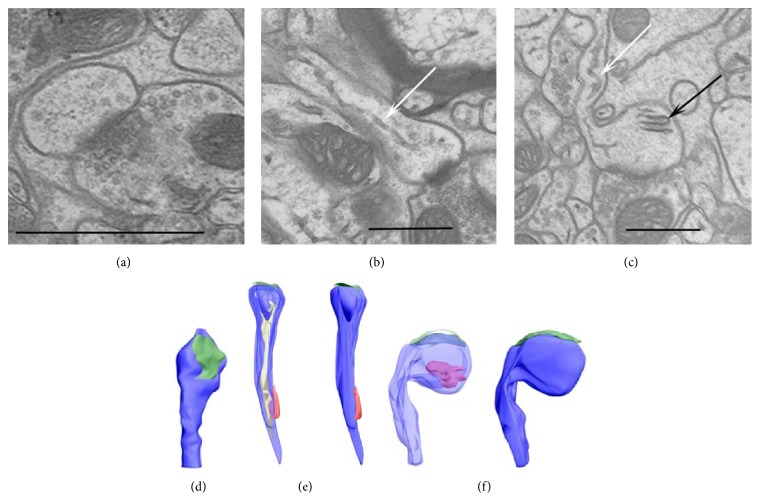
3D serial section EM reconstruction of three spine types from B2 barrel hollow, also shown in single electron micrographs: sER-free spine (a), spine containing sER (b), and spine containing spine apparatus (c). White arrows indicate sER ((b) and (c)) and black arrow indicates spine apparatus (c). (d)–(f) show reconstruction of dendritic spines (blue): excitatory synapses (green), inhibitory synapse (red; only (e)), smooth endoplasmic reticulum (yellow; (e)), and spine apparatus (red; (f)). Scale bars: 0.5 *μ*m.

Dendritic spines were classified into three categories according to their content: sER-free spines (Figures 1(a) and 1(d)), spines containing sER only (Figures 1(b) and 1(e)), and spines containing SA (Figures 1(c) and 1(f)). The sER was visible as membranous cisternae inside the spines (Figure 1(b)). The SA was identified as an array of membranous cisternae interleaved with electron-dense plates (Figure 1(c)), as described by Ostroff et al. [4].

#### 3.2.1. Density of Single-Synapse Spines

**Figure 2:**
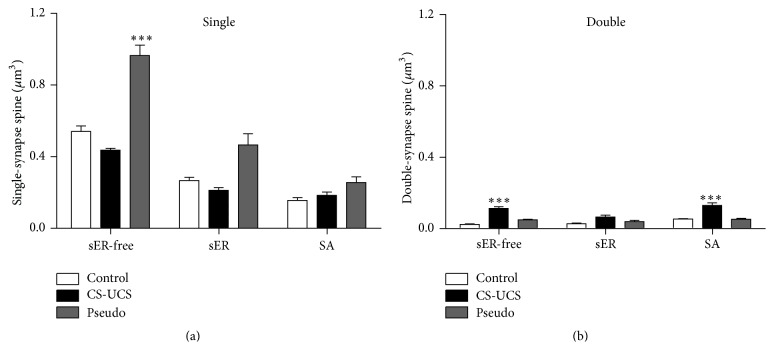
Density of single- (a) and double-synapse spines (b): sER-free, containing sER, and containing spine apparatus (SA). The graphs show means ± SEM (one-way ANOVA with* post hoc* Tukey's test, ^*∗∗∗*^ *P* < 0.001).

The density of sER-free single-synapse spines increased approximately twofold after pseudoconditioning (pseudoconditioned group: 0.96 ± 0.08/*μ*m^3^; control group: 0.54 ± 0.04/*μ*m^3^; *F*_(1, 44)_ = 29.12, *P* < 0.0001) but did not show any significant change after conditioning (0.44 ± 0.01/*μ*m^3^; Figure 2(a)). In the conditioned and pseudoconditioned animals, the mean densities of single-synapse spines containing sER and SA did not significantly change (sER: 0.21 ± 0.02/*μ*m^3^ and 0.47 ± 0.09/*μ*m^3^, SA: 0.18 ± 0.03/*μ*m^3^ and 0.26 ± 0.04/*μ*m^3^, resp.) compared to control group (sER: 0.27 ± 0.03/*μ*m^3^, SA: 0.16 ± 0.02/*μ*m^3^; Figure 2(a)).

#### 3.2.2. Density of Double-Synapse Spines

A twofold increase and a fourfold increase in the density of double-synapse spines containing SA and sER-free spines, respectively, were found in the conditioned animals (SA: 0.13 ± 0.02/*μ*m^3^, sER-free: 0.11 ± 0.01/*μ*m^3^) when compared with control animals (SA: 0.05 ± 0.004/*μ*m^3^, sER-free: 0.02 ± 0.005/*μ*m^3^; *F*_(1, 44)_ = 11.26, *P* < 0.0001), whereas statistically significant differences were not observed between the pseudoconditioned (SA: 0.05 ± 0.006/*μ*m^3^, sER-free: 0.05 ± 0.005/*μ*m^3^) and control mice (Figure 2(b)). The density of spines containing sER was the highest in the conditioned group, but differences between the groups failed to reach significance (control: 0.03 ± 0.005/*μ*m^3^; conditioned: 0.06 ± 0.01/*μ*m^3^; pseudoconditioned: 0.04 ± 0.01/*μ*m^3^; Figure 2(b)).

### 3.3. Morphological Analysis of Spines

Results of the measurements are presented in supplementary Table 1 in Supplementary Material available online at http://dx.doi.org/10.1155/2016/9828517.

#### 3.3.1. Area of Postsynaptic Density (PSD)

**Figure 3:**
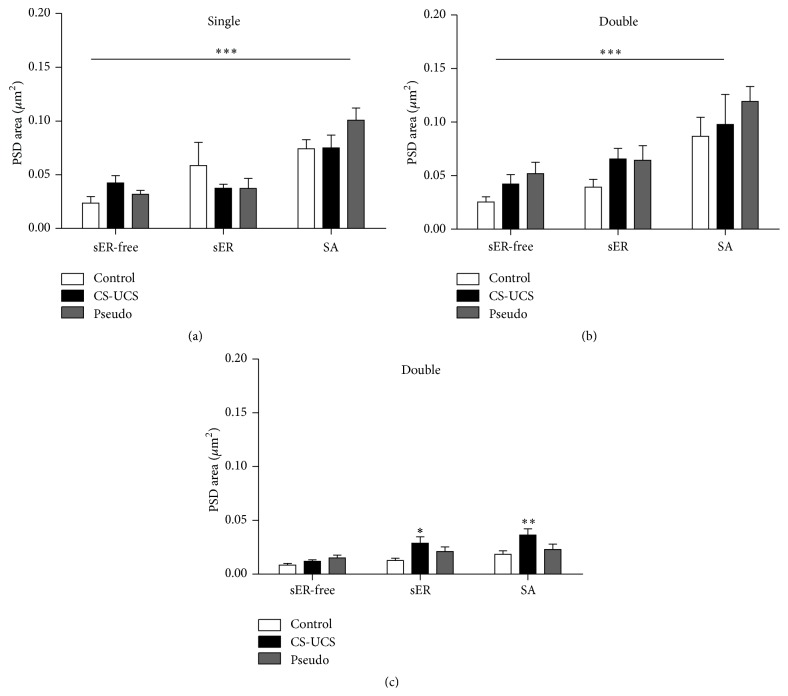
PSD area of excitatory ((a) and (b)) and inhibitory (c) synapses of single- and double-synapse spines: sER-free, containing sER, and containing SA. The graphs show means ± SEM (two-way ANOVA with* post hoc* Bonferroni test; ^*∗∗∗*^ *P* < 0.001, ^*∗∗*^ *P* < 0.01, and ^*∗*^ *P* < 0.05).

*PSD of Excitatory Synapses.* In all groups, single- and double-synapse spines containing SA had larger PSD area of excitatory synapses as compared to sER-free spines and spines containing sER in the same group of animals (Figures 3(a) and 3(b)). sER-free spines had similar PSD area of excitatory synapses in the control, conditioned, and pseudoconditioned animals. Similarly, spines containing sER had the same PSD area of excitatory synapses in control, conditioned, and pseudoconditioned animals.

*PSD of Inhibitory Synapses.* Conditioning induced a significant increase in the PSD area of inhibitory synapses located on double-synapse spines containing sER and SA (Figure 3(c)). The PSD area in sER-free spines did not significantly change in conditioned and pseudoconditioned animals as compared with the control group.

#### 3.3.2. Volume and Location of Spine Apparatus in Dendritic Spines

**Figure 4:**
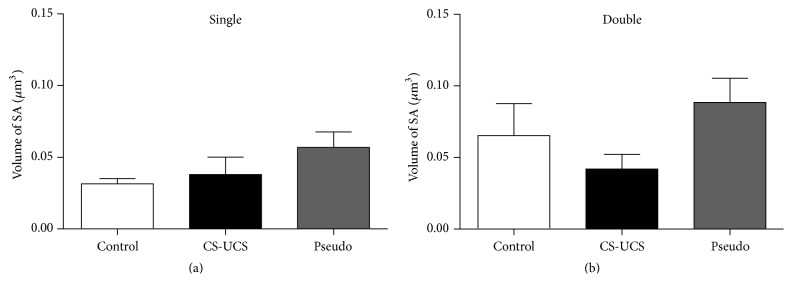
Volume of spine apparatus in single- (a) and double-synapse spines (b). The graphs show means ± SEM (one-way ANOVA with* post hoc* Tukey's test).

The SA usually included 2–6 cisternae and conditioning did not influence that number. No differences were also observed in the volume of SA between the groups of animals (Figures 4(a) and 4(b)).

The vast majority of SA (single: 56.67%; double: 63.33%) was located on the border between spine head and neck and this location did not change after conditioning or pseudoconditioning.

#### 3.3.3. Occurrence of Polyribosomes in Dendritic Spines

**Figure 5:**
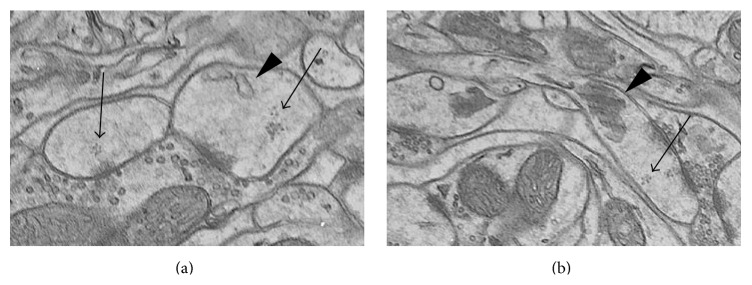
Electron micrographs showing polyribosomes in sER-free, sER-containing (a), and SA-containing (b) spines. Arrows: polyribosomes; arrowhead: sER (a) and spine apparatus (b).

Polyribosomes (Figure 5) were identified as described previously [7] and the mean number of polyribosomes per spine was assessed. Almost all single- and double-synapse spines (96.67%) containing spine apparatus also contained polyribosomes in all experimental groups.

**Figure 6:**
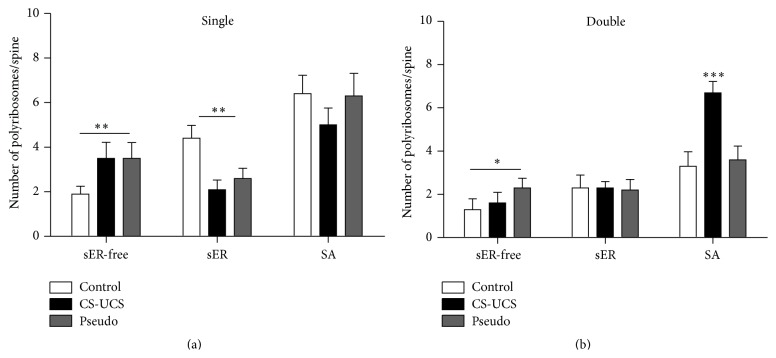
Number of polyribosomes in the single- (a) and double-synapse spines (b): sER-free, containing sER, and containing SA. The graphs show means ± SEM (chi square test and one-way ANOVA with* post hoc* Tukey's test: ^*∗∗∗*^ *P* < 0.001, ^*∗∗*^ *P* < 0.01, and ^*∗*^ *P* < 0.05).

Polyribosomes located in the single-synapse spines were more frequent in the sER-free spines of conditioned and pseudoconditioned animals, and, conversely, the density of polyribosomes located in single-synapse spines containing sER decreased after training. There were no changes in the number of polyribosomes located in single-synapse spines containing SA (Figure 6(a)). Conditioning increased the number of polyribosomes located in double-synapse spines containing SA, while pseudoconditioning increased the number of polyribosomes located in the sER-free spines. There were no changes in the number of polyribosomes located in double-synapse spines containing sER (Figure 6(b)).

### 3.4. Shape of Spines

**Figure 7:**
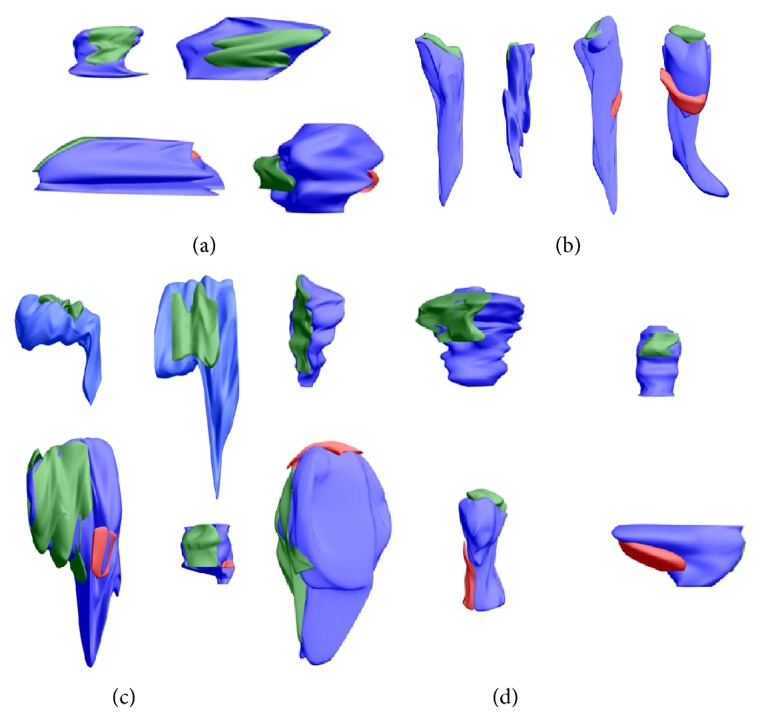
3D reconstruction of single- and double-synapse spines from serial micrographs showing different shapes of spines: stubby (a), thin (b), mushroom (c), and intermediate (d). Color areas as in Figure 1.

**Figure 8:**
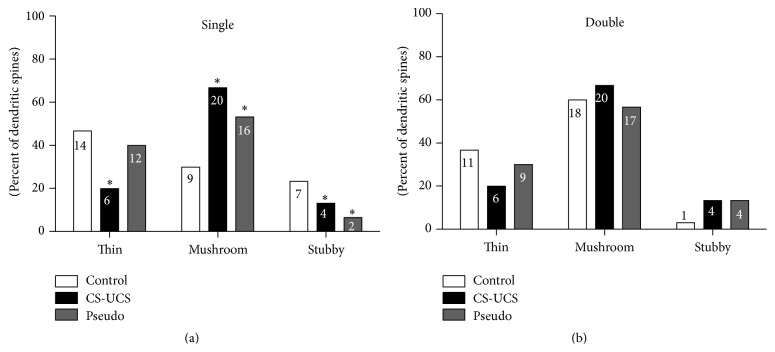
Shapes of single- (a) and double-synapse spines (b): thin, mushroom, and stubby. The graphs show percentages of spine types and their numbers inside the bars (chi square test: ^*∗*^ *P* < 0.05).

Among 60 spines selected for reconstruction (Figure 7), in the control group, the rarest were stubby spines (Figure 7(a)), constituting 23.33% of single-synapse spines and only 3.33% of double-synapse spines. The proportions of other spine shapes were also dependent on type of spine. Single-synapse spines showed predominance of thin spines (Figure 7(b)) that constituted almost half of all such spines (46.67%), while among double-synapse spines about one-third were thin spines (36.67%). Mushroom-shaped spines (Figure 7(c)) accounted for 30% of single-synapse spines and for 60% of double-synapse spines (Figure 8).

The majority of stubby spines were sER-free (single: 57.14%; double: 100%). Thin spines mostly contained sER (single: 42.86%; double: 54.55), while mushroom spines predominantly contained SA (single: 66.67%; double: 44.44%).

Conditioning and pseudoconditioning induced an increase in the proportion of mushroom single-synapse spines at the expense of stubby and thin (only conditioning) single-synapse spines (*χ* ^2^_(4)_ = 10.31, *P* = 0.0356; Figure 8(a)). There were no experience-dependent changes in shapes of double-synapse spines (*χ* ^2^_(4)_ = 3.716, *P* = 0.4458).

We also observed intermediate shapes of spines [33]: short thin spines (the length of spine being 2-3 times longer than the diameter of neck and similar diameters of head and neck) or stubby-mushroom spines (the diameter of spine head being 1.5–2.5 times bigger than the diameter of neck and the length of spine being about 1.5–2.5 times longer than the diameter of neck) (Figure 7(d)). Intermediate spines were rare (3.89% of all spines).

Results of the study are summarized in Table 1.

## 4. Discussion

This study presents for the first time the effect of fear conditioning on a broad range of morphological parameters of dendritic spines in the somatosensory cortex. Some of its results obtained in control animals confirm findings reported from other areas of the central nervous system, demonstrating that the spines containing spine apparatus (SA) are mostly mushroom-shaped [4, 15, 34]—in the barrel cortex about 80% of double-synapse spines and 77% of single—synapse spines—and that they almost always contain polyribosomes [4]. However, proportions of spine shapes found in the somatosensory cortex differ from that observed in other brain regions; for example, in the hippocampus of adult rat, about 65% of spines were thin, 25% were mushroom, and only about 10% were stubby spines [15]. These apparent differences might reflect differences in spine types in different brain regions.

The percentage of sER-free spines, spines containing sER, and spines containing SA is similar in all analyzed groups of animals. In the lateral amygdala, about 20% spines contained SA, and approximately 10% spines contained sER but not SA [4], while in the hippocampus, depending on different location, from 14% to 37% spines expressed synaptopodin, a marker of SA [27]. The above results are the most consistent with our findings in somatosensory cortex, where about 20% of single-synapse spines and almost a half of double-synapse spines constituting only about 10% of all spines [2, 12] contained SA. Our 3D reconstruction analysis of spine shapes showed that mushroom spines mostly contained spine apparatus and thin spines mostly contained sER, while stubby spines were predominantly sER-free.

We have found that associative fear learning differentially regulates the density of single- and double-synapse spines and exerts a more profound effect on the latter spines. An increase in density was observed in case of sER-free and SA-containing double-synapse spines in somatosensory cortex always bearing an excitatory and an inhibitory synapse [2, 12, 35]. There were no changes, however, in the density of single-synapse spines, associated exclusively with excitatory synapses. Other effects of learning on double-synapse spines included an increase in PSD area of inhibitory synapses in spines containing sER and SA as well as an increase in the number of polyribosomes in SA-containing spines. Single-synapse spines responded to conditioning by a decrease in the number of thin spines.

These findings are consistent with the results of our previous studies demonstrating conditioning-induced formation of inhibitory synapses on double-synapse spines in the barrel cortex [3] and increase in the density of polyribosomes associated with both excitatory and inhibitory synapses located on dendritic spines [7]. The present study shows that the learning-associated changes of double-synapse spines mainly involve sER-free and SA-containing spines. The sER-free spines are the smallest and are considered to be the most unstable, transient spines with the highest motility and very short half-life* in vivo* [17, 23, 28], whereas the spines containing SA are the largest spines; they form stable synaptic connections [23]. These spines have larger PSD areas, as also observed in the present study, and more numerous AMPA glutamate receptors, which could enhance the strength of their synapses [28, 36, 37], and they contain more frequent polyribosomes, suggesting local protein synthesis [4, 26]. Hence, in case of double-synapse spines, associative learning seems to act bidirectionally: it temporarily enhances learning capacity by adding sER-free transient spines and promotes transformation of preexisting spines into the most stable SA-containing spines to stabilize connectivity.

Ostroff et al. [4] proposed that SA may be required for the induction of local translation or for posttranslational protein changes. Memory formation seems to involve strengthening and stabilization of synapses requiring newly produced proteins. In the barrel cortex, conditioning upregulates the local protein synthesis in both single-synapse spines and double-synapse spines but this effect seems to be more effective in double-synapse spines containing SA, as it is accompanied by increase in PSD area. Hence, double-synapse spines are the preferential candidates for participation in learning-associated memory pathways in the barrel cortex.

Learning-induced increase in the number of polyribosomes in single-synapse spines is accompanied by enhanced frequency of mushroom-shaped spines but not of spines containing SA. On the other hand, in double-synapse spines, the response to conditioning includes increased incidence of SA but not increase in the number of mushroom spines. In a previous study we suggested that during conditioning inhibitory inputs could be added to preexisting single-synapse spines [3]. Now, we propose that only those single-synapse spines, which undergo special “preparation” during the learning process, including increase in the density of polyribosomes, increased PSD areas of excitatory synapses, and the spine shapes changing toward mushroom spines, are ready for the acquisition of new inhibitory synapses and formation of SA, which might complete their transformation into “memory spines.”

These observations suggest that differential regulation of single- and double-synapse spines by associative learning might involve local synthesis of proteins participating in shape remodeling of single-synapse spines and involved in formation of SA in double-synapse spines.

Other morphological parameters of dendritic spines did not seem to be influenced by learning, neither total spine area (results not shown) nor location of SA and its volume.

We also used the pseudoconditioned group to test whether the observed changes were directly associated with the influence of learning or resulted only from the random application of two kinds of sensory stimuli. Pseudoconditioning is believed to induce a general sensitization of the animal to the conditioned stimulus [38]. Some effects observed in this study (increase in the number of polyribosomes in sER-free spines, decrease in the number of polyribosomes in sER-containing spines, increase in the number of mushroom spines, and decrease in the number of stubby spines) were observed after both, conditioning and pseudoconditioning, so they should be attributed to such sensitization. However, pseudoconditioning alone brought about a significant increase in the density of sER-free single-synapse spines. Such effects of pseudoconditioning alone were only occasionally reported. Cybulska-Klosowicz and Kossut [38] observed that pseudoconditioning activated the contralateral and ipsilateral barrel field, in contrast to a decrease in bilateral activation seen in the conditioned groups. In our previous studies on the barrel cortex we found pseudoconditioning-induced increase in the density of single-synapse spines [3] and a decrease in the density of polyribosomes in dendritic shafts not associated with synapses [7]. The explanation of a sole effect of pseudoconditioning can only be speculative: in contrast to the situation in which an animal learns a sequence of events, a random application of an unpleasant stimulus seems to induce some kind of stress, influencing brain plasticity in a different manner. The present finding suggests that this effect mainly concerns the smallest and most transient spines.

## 5. Conclusions

Results of the present study demonstrate that associative fear learning produces different effects on single- and double-synapse spines in the barrel cortex: it promotes maturation and stabilization of double-synapse spines, which might possibly contribute to permanent memory formation, and upregulates protein synthesis in single-synapse spines, which might prepare them to accept new inhibitory synapses and transform into double-synapse spines.

## Supplementary Material

Supplementary material contains numerical results of spine parameter measurements: volume of spine apparatus, number of polyribosomes per spine, spine length, diameter of head and neck, area of postsynaptic density.

## Figures and Tables

**Table 1:** Statistically significant effects of conditioning and pseudoconditioning on dendritic spines in the barrel cortex.

	Single-synapse spines	Double-synapse spines
Effects after conditioning	Decrease in the number of thin spines	Increase in the number of sER-free and SA-containing spines Increase in inhibitory PSD area in sER- and SA-containing spines Increase in the number of polyribosomes in SA-containing spines

Effects after conditioning and pseudoconditioning	Increase in the number of polyribosomes in sER-free spines Decrease in the number of polyribosomes in sER-containing spines Increase in the number of mushroom spines Decrease in the number of stubby spines	

Effects after pseudoconditioning only	Increase in the number of sER-free spines	Increase in the number of polyribosomes in sER-free spines

## References

[1] Micheva K. D., Beaulieu C. (1995). An anatomical substrate for experience-dependent plasticity of the rat barrel field cortex. *Proceedings of the National Academy of Sciences of the United States of America*.

[2] Knott G. W., Quairiaux C., Genoud C., Welker E. (2002). Formation of dendritic spines with GABAergic synapses induced by whisker stimulation in adult mice. *Neuron*.

[3] Jasinska M., Siucinska E., Cybulska-Klosowicz A. (2010). Rapid, learning-induced inhibitory synaptogenesis in murine barrel field. *Journal of Neuroscience*.

[4] Ostroff L. E., Cain C. K., Bedont J., Monfils M. H., LeDoux J. E. (2010). Fear and safety learning differentially affect synapse size and dendritic translation in the lateral amygdala. *Proceedings of the National Academy of Sciences of the United States of America*.

[5] Lushnikova I., Skibo G., Muller D., Nikonenko I. (2011). Excitatory synaptic activity is associated with a rapid structural plasticity of inhibitory synapses on hippocampal CA1 pyramidal cells. *Neuropharmacology*.

[6] Bourne J. N., Harris K. M. (2011). Coordination of size and number of excitatory and inhibitory synapses results in a balanced structural plasticity along mature hippocampal CA1 dendrites during LTP. *Hippocampus*.

[7] Jasinska M., Siucinska E., Jasek E., Litwin J. A., Pyza E., Kossut M. (2013). Fear learning increases the number of polyribosomes associated with excitatory and inhibitory synapses in the barrel cortex. *PLoS ONE*.

[8] Siucinska E., Kossut M. (1996). Short-lasting classical conditioning induces reversible changes of representational maps of vibrissae in mouse SI cortex—a 2DG study. *Cerebral Cortex*.

[9] Skibinska A., Lech M., Kossut M. (2005). Differential regulation of cortical NMDA receptor subunits by sensory learning. *Brain Research*.

[10] Gierdalski M., Jablonska B., Siucinska E., Lech M., Skibinska A., Kossut M. (2001). Rapid regulation of GAD67 mRNA and protein level in cortical neurons after sensory learning. *Cerebral Cortex*.

[11] Siucinska E., Kossut M., Stewart M. G. (1999). GABA immunoreactivity in mouse barrel field after aversive and appetitive classical conditioning training involving facial vibrissae. *Brain Research*.

[12] Jasińska M., Siucińska E., Głazewski S., Pyza E., Kossut M. (2006). Characterization and plasticity of the double synapse spines in the barrel cortex of the mouse. *Acta Neurobiologiae Experimentalis*.

[13] Popov V. I., Davies H. A., Rogachevsky V. V. (2004). Remodelling of synaptic morphology but unchanged synaptic density during late phase long-term potentiation (LTP): a serial section electron micrograph study in the dentate gyrus in the anaesthetised rat. *Neuroscience*.

[14] Harris K. M., Fiala J. C., Ostroff L. (2003). Structural changes at dendritic spine synapses during long-term potentiation. *Philosophical Transactions of the Royal Society B: Biological Sciences*.

[15] Harris K. M., Jensen F. E., Tsao B. (1992). Three-dimensional structure of dendritic spines and synapses in rat hippocampus (CA1) at postnatal day 15 and adult ages: implications for the maturation of synaptic physiology and long-term potentiation. *Journal of Neuroscience*.

[16] Spacek J., Harris K. M. (1997). Three-dimensional organization of smooth endoplasmic reticulum in hippocampal CA1 dendrites and dendritic spines of the immature and mature rat. *Journal of Neuroscience*.

[17] Kasai H., Matsuzaki M., Noguchi J., Yasumatsu N., Nakahara H. (2003). Structure-stability-function relationships of dendritic spines. *Trends in Neurosciences*.

[18] Holtmaat A. J. G. D., Trachtenberg J. T., Wilbrecht L. (2005). Transient and persistent dendritic spines in the neocortex *in vivo*. *Neuron*.

[19] Trachtenberg J. T., Chen B. E., Knott G. W. (2002). Long-term in vivo imaging of experience-dependent synaptic plasticity in adult cortex. *Nature*.

[20] Gray E. G. (1959). Electron microscopy of synaptic contacts on dendrite spines of the cerebral cortex. *Nature*.

[21] Deller T., Merten T., Roth S. U., Mundel P., Frotscher M. (2000). Actin-associated protein synaptopodin in the rat hippocampal formation: localization in the spine neck and close association with the spine apparatus of principal neurons. *Journal of Comparative Neurology*.

[22] Fifková E., Markham J. A., Delay R. J. (1983). Calcium in the spine apparatus of dendritic spines in the dentate molecular layer. *Brain Research*.

[23] Vlachos A., Korkotian E., Schonfeld E., Copanaki E., Deller T., Segal M. (2009). Synaptopodin regulates plasticity of dendritic spines in hippocampal neurons. *The Journal of Neuroscience*.

[24] Korkotian E., Frotscher M., Segal M. (2014). Synaptopodin regulates spine plasticity: mediation by calcium stores. *Journal of Neuroscience*.

[25] Steward O., Reeves T. M. (1988). Protein-synthetic machinery beneath postsynaptic sites on CNS neurons: association between polyribosomes and other organelles at the synaptic site. *The Journal of Neuroscience*.

[26] Pierce J. P., Mayer T., McCarthy J. B. (2001). Evidence for a satellite secretory pathway in neuronal dendritic spines. *Current Biology*.

[27] Deller T., Orth C. B., Del Turco D. (2007). A role for synaptopodin and the spine apparatus in hippocampal synaptic plasticity. *Annals of Anatomy*.

[28] Bourne J., Harris K. M. (2007). Do thin spines learn to be mushroom spines that remember?. *Current Opinion in Neurobiology*.

[29] Jedlicka P., Vlachos A., Schwarzacher S. W., Deller T. (2008). A role for the spine apparatus in LTP and spatial learning. *Behavioural Brain Research*.

[30] Deller T., Korte M., Chabanis S. (2003). Synaptopodin-deficient mice lack a spine apparatus and show deficits in synaptic plasticity. *Proceedings of the National Academy of Sciences of the United States of America*.

[31] Fiala J. C., Harris K. M. (2001). Extending unbiased stereology of brain ultrastructure to three-dimensional volumes. *Journal of the American Medical Informatics Association*.

[32] Ostroff L. E., Fiala J. C., Allwardt B., Harris K. M. (2002). Polyribosomes redistribute from dendritic shafts into spines with enlarged synapses during LTP in developing rat hippocampal slices. *Neuron*.

[33] Arellano J. I., Benavides-Piccione R., Defelipe J., Yuste R. (2007). Ultrastructure of dendritic spines: correlation between synaptic and spine morphologies. *Frontiers in Neuroscience*.

[34] Kuwajima M., Spacek J., Harris K. M. (2013). Beyond counts and shapes: Studying pathology of dendritic spines in the context of the surrounding neuropil through serial section electron microscopy. *Neuroscience*.

[35] Jones E. G., Powell T. P. (1969). Morphological variations in the dendritic spines of the neocortex. *Journal of Cell Science*.

[36] Malenka R. C., Nicoll R. A. (1999). Long-term potentiation—a decade of progress?. *Science*.

[37] Matsuzaki M., Ellis-Davies G. C. R., Nemoto T., Miyashita Y., Iino M., Kasai H. (2001). Dendritic spine geometry is critical for AMPA receptor expression in hippocampal CA1 pyramidal neurons. *Nature Neuroscience*.

[38] Cybulska-Klosowicz A., Kossut M. (2006). Early-phase of learning enhances communication between brain hemispheres. *European Journal of Neuroscience*.

