# Supplementary material for: Effect of Associative Learning on Memory Spine Formation in Mouse Barrel Cortex
Source: Neural Plast. 2015 Dec 24;2016:9828517. doi: 10.1155/2016/9828517 (PMC4706958; doi:10.1155/2016/9828517)
Supplement: Supplementary file 1 — Supplementary material contains numerical results of spine parameter measurements: volume of spine apparatus, number of polyribosomes per spine, spine length, diameter of head and neck, area of postsynaptic density. [file 9828517.f1.pdf]

Supplementary Table 1

Results of spine parameter measurements

	single-synapse spines			double-synapse spines		
**parameter**	**mean ± SEM**	**F/ t/ χ^2**	**P**	**mean ± SEM**	**F/ t/ χ^2**	**P**
Volume of SA [μm^3]	C: 0.031 ± 0.004<br>Cd: 0.038 ± 0.012<br>P: 0.057 ± 0.011	F = 1.86	0.17	C: 0.065 ± 0.022<br>Cd: 0.042 ± 0.010<br>P: 0.088 ± 0.017	F = 1.83	0.18
Number of polyribosomes per sER-free spine	C: 1.9 ± 0.35<br>Cd: 3.5 ± 0.72<br>P: 3.5 ± 0.70	F = 6.14<br>χ^2 = 15.08	<0.0001<br><br>< 0.001<br>< 0.001	C: 1.3 ± 0.49<br>Cd: 1.6 ± 0.50<br>P: 2.3 ± 0.45	F = 9.40<br>χ^2 = 9.79	<0.0001<br><br>> 0.05<br>< 0.05
Number of polyribosomes per sER-containing spine	C: 4.4 ± 0.58<br>Cd: 2.1 ± 0.43<br>P: 2.6 ± 0.45		< 0.001<br>< 0.001	C: 2.3 ± 0.60<br>Cd: 2.3 ± 0.30<br>P: 2.2 ± 0.49		> 0.05<br>> 0.05
Number of polyribosomes per SA-containing spine	C: 6.4 ± 0.83<br>Cd: 5.0 ± 0.76<br>P: 6.3 ± 1.01		> 0.05<br>> 0.05	C: 3.3 ± 0.67<br>Cd: 6.7 ± 0.52<br>P: 3.6 ± 0.64		< 0.001<br>> 0.05
Length of sER-free spine [μm]	C: 0.54 ± 0.04<br>Cd: 0.64 ± 0.09<br>P: 0.76 ± 0.04	F = 9.24<br><br>t_{CD} = 0.00<br>t_P = 0.90	0.0002<br><br>> 0.05<br>> 0.05	C: 0.68 ± 0.05<br>Cd: 0.59 ± 0.10<br>P: 0.59 ± 0.05	F = 4.97<br><br>t_{CD} = 0.92<br>t_P = 0.92	0.009<br><br>> 0.05<br>> 0.05
Length of sER-containing spine [μm]	C: 0.54 ± 0.05<br>Cd: 0.56 ± 0.06<br>P: 0.63 ± 0.05	t_{CD} = 0.99<br>t_P = 0.49	> 0.05<br>> 0.05	C: 0.60 ± 0.07<br>Cd: 0.58 ± 0.03<br>P: 0.63 ± 0.07	t_{CD} = 0.14<br>t_P = 0.35	> 0.05<br>> 0.05
Length of SA-containing spine [μm]	C: 0.62 ± 0.04<br>Cd: 0.60 ± 0.07<br>P: 0.90 ± 0.06	t_{CD} = 1.48<br>t_P = 1.73	> 0.05<br>> 0.05	C: 0.80 ± 0.09<br>Cd: 0.66 ± 0.06<br>P: 0.84 ± 0.07	t_{CD} = 1.41<br>t_P = 0.42	> 0.05<br>> 0.05
Diameter of sER-free spine head [μm]	C: 0.43 ± 0.04<br>Cd: 0.53 ± 0.07<br>P: 0.41 ± 0.04	F = 1.83<br><br>t_{CD} = 0.42<br>t_P = 0.10	0.17<br><br>> 0.05<br>> 0.05	C: 0.44 ± 0.04<br>Cd: 0.54 ± 0.08<br>P: 0.39 ± 0.03	F = 112.1<br><br>t_{CD} = 2.52<br>t_P = 1.10	< 0.0001<br><br>< 0.05<br>> 0.05
Diameter of sER-containing spine head [μm]	C: 0.41 ± 0.04<br>Cd: 0.66 ± 0.10<br>P: 0.48 ± 0.05	t_{CD} = 1.09<br>t_P = 0.29	> 0.05<br>> 0.05	C: 0.49 ± 0.06<br>Cd: 0.61 ± 0.18<br>P: 0.48 ± 0.05	t_{CD} = 2.89<br>t_P = 0.32	< 0.05<br>> 0.05

Diameter of SA-containing spine head [µm]	C: 0.62 ± 0.08<br>Cd: 0.82 ± 0.15<br>P: 0.68 ± 0.05	$t_{CD} = 0.84$<br>$t_{P} = 0.27$	> 0.05<br>> 0.05	C: 0.73 ± 0.05<br>Cd: 0.96 ± 0.13<br>P: 0.68 ± 0.08	$t_{CD} = 5.75$<br>$t_{P} = 1.03$	< 0.01<br>> 0.05
Diameter of sER-free spine neck [µm]	C: 0.27 ± 0.04<br>Cd: 0.27 ± 0.03<br>P: 0.22 ± 0.02	F = 0.40<br>$t_{CD} = 0.07$<br>$t_{P} = 1.15$	0.67<br>> 0.05<br>> 0.05	C: 0.24 ± 0.03<br>Cd: 0.30 ± 0.03<br>P: 0.23 ± 0.04	F = 1.11<br>$t_{CD} = 1.24$<br>$t_{P} = 0.21$	0.33<br>> 0.05<br>> 0.05
Diameter of sER-containing spine neck [µm]	C: 0.24 ± 0.03<br>Cd: 0.29 ± 0.04<br>P: 0.24 ± 0.03	$t_{CD} = 0.99$<br>$t_{P} = 0.09$	> 0.05<br>> 0.05	C: 0.25 ± 0.04<br>Cd: 0.29 ± 0.05<br>P: 0.24 ± 0.04	$t_{CD} = 0.73$<br>$t_{P} = 0.23$	> 0.05<br>> 0.05
Diameter of SA-containing spine neck [µm]	C: 0.27 ± 0.02<br>Cd: 0.29 ± 0.04<br>P: 0.26 ± 0.02	$t_{CD} = 0.35$<br>$t_{P} = 0.38$	> 0.05<br>> 0.05	C: 0.29 ± 0.02<br>Cd: 0.32 ± 0.03<br>P: 0.27 ± 0.03	$t_{CD} = 0.54$<br>$t_{P} = 0.46$	> 0.05<br>> 0.05
Excitatory PSD area of sER-free spine [µm^2]	C: 0.024 ± 0.006<br>Cd: 0.043 ± 0.027<br>P: 0.032 ± 0.003	F = 19.58<br>$t_{CD} = 1.28$<br>$t_{P} = 0.57$	< 0.0001<br>> 0.05<br>> 0.05	C: 0.026 ± 0.005<br>Cd: 0.042 ± 0.009<br>P: 0.052 ± 0.010	F = 15.00<br>$t_{CD} = 0.82$<br>$t_{P} = 1.31$	< 0.0001<br>> 0.05<br>> 0.05
Excitatory PSD area of sER-containing spine [µm^2]	C: 0.059 ± 0.021<br>Cd: 0.038 ± 0.037<br>P: 0.037 ± 0.009	$t_{CD} = 1.44$<br>$t_{P} = 1.45$	> 0.05<br>> 0.05	C: 0.039 ± 0.007<br>Cd: 0.066 ± 0.009<br>P: 0.064 ± 0.013	$t_{CD} = 1.31$<br>$t_{P} = 1.24$	> 0.05<br>> 0.05
Excitatory PSD area of SA-containing spine [µm^2]	C: 0.074 ± 0.008<br>Cd: 0.075 ± 0.012<br>P: 0.100 ± 0.011	$t_{CD} = 0.07$<br>$t_{P} = 1.81$	> 0.05<br>> 0.05	C: 0.087 ± 0.017<br>Cd: 0.098 ± 0.027<br>P: 0.119 ± 0.014	$t_{CD} = 0.55$<br>$t_{P} = 1.61$	> 0.05<br>> 0.05
Inhibitory PSD area of sER-free spine [µm^2]				C: 0.009 ± 0.001<br>Cd: 0.012 ± 0.001<br>P: 0.015 ± 0.003	F = 7.43<br>$t_{CD} = 0.60$<br>$t_{P} = 1.12$	0.0011<br>> 0.05<br>> 0.05
Inhibitory PSD area of sER-containing spine [µm^2]				C: 0.013 ± 0.002<br>Cd: 0.029 ± 0.006<br>P: 0.021 ± 0.004	$t_{CD} = 2.87$<br>$t_{P} = 1.47$	< 0.05<br>> 0.05
Inhibitory PSD area of SA-containing spine [µm^2]				C: 0.019 ± 0.003<br>Cd: 0.037 ± 0.006<br>P: 0.023 ± 0.005	$t_{CD} = 3.21$<br>$t_{P} = 0.79$	< 0.01<br>> 0.05

C – control mice; Cd – conditioned mice; P – pseudoconditioned mice
